# Trends in Mixed Pain Research Over Three Decades (1993–2024): A Bibliometric Analysis

**DOI:** 10.1007/s11916-025-01371-6

**Published:** 2025-03-19

**Authors:** Matteo Luigi Giuseppe Leoni, Marco Mercieri, Roberto Gazzeri, Marco Cascella, Martina Rekatsina, Omar Viswanath, Alberto Pasqualucci, Giustino Varrassi

**Affiliations:** 1 Department of Medical and Surgical Sciences and Translational Medicine, Sapienza University of Rome, Rome, Italy; 2 Pain Management Unit, San Giovanni-Addolorata Hospital, Rome, Italy; 3 Department of Medicine, Surgery and Dentistry, University of Salerno, Baronissi, 84081 Italy; 4 Department of Anesthesiology and Pain Medicine, National and Kapodistrian University of Athens, Athens, Greece; 5 Department of Anesthesiology, Creighton University School of Medicine, Phoenix, AZ USA; 6 Mountain View Headache and Spine Institute, Phoenix, AZ USA; 7 Anaesthesia and Critical Care, University of Perugia, Perugia, Italy; 8 Fondazione Paolo Procacci, Rome, 00193 Italy

**Keywords:** Mixed pain, Bibliometric analysis, Neuropathic pain, Nociceptive pain, Nociplastic pain, Pain management, Quality of life, Multidisciplinary approach

## Abstract

**Background:**

The term “mixed pain” is frequently used in clinical practice to describe the coexistence of nociceptive, neuropathic, and nociplastic pain mechanisms. However, its inconsistent use and lack of a formal definition warrant further investigation. This bibliometric analysis aims to explore publication trends, research networks, and key themes in mixed pain literature.

**Methods:**

A bibliometric analysis was conducted using the Web of Science Core Collection. The search was performed in February 2024, with journal rankings obtained from Journal Citation Reports™ 2022 (Clarivate Analytics). Extracted data included publication trends, citation analysis, co-authorship networks, and keyword mapping.

**Results:**

A total of 229 publications were identified, demonstrating an increasing trend in both publication volume and citations. Most studies were published in high-ranking Q1 journals as research (77%) and review articles (19%). The USA (21%), Italy (15%), and Germany (12%) were the leading contributors, yet global collaboration was weak, with limited co-authorship connections except within the USA. The keyword analysis revealed five major research clusters, with “neuropathic pain,” “management,” and “quality of life” emerging as central themes.

**Conclusions:**

Despite the progressive increase in mixed pain articles in highly ranked journals, this bibliometric analysis highlighted the absence of a well-structured collaborative network among authors and a lack of clear connections between keywords. Given the critical clinical implications of mixed pain, further high-quality studies on this topic and enhanced international collaborations are recommended.

## Background

The definition of pain was recently updated by the International Association for the Study of Pain (IASP), and refined as “An unpleasant sensory and emotional experience associated with, or resembling that associated with, actual or potential tissue damage” [1]. This new definition acknowledges the complex nature of pain, recognizing not only its physical but also its emotional dimensions. In fact, pain can arise in conditions where tissue damage is potential or even actually absent. Starting from these elements, a new mechanism was recently introduced by the IASP: the nociplastic pain [2]. This concept was introduced to better describe the type of pain that arises from altered nociception, even in the absence of clear evidence of actual or threatened tissue damage. Moreover, in the pathophysiological classification of pain, the IASP also defines neuropathic pain as a clinical condition that requires evidence of a demonstrable lesion or disease affecting the somatosensory nervous system [3]. In contrast, nociceptive pain, often regarded as the most common type of pain in humans, arises from actual or potential harm to tissues not involving the nervous system [4]. This type of pain is triggered by the activation of nociceptors.

However, the distinct pathophysiological classification of pain into purely neuropathic, nociceptive, or nociplastic categories has often been criticized for being both imprecise and overly simplistic [5, 6]. In clinical practice, it is common to encounter patients who exhibit an overlap of these three pain mechanisms (neuropathic, nociceptive, and nociplastic). To describe this condition, the term “mixed pain” is frequently used and accepted by pain clinicians [7–10], despite it not being formally recognized in the IASP taxonomy and lacking an official definition. Additionally, it is important to note that the nociplastic component is not always present in every case of mixed pain [11]. The diagnosis of mixed pain is clinical and relies on the careful assessment of symptoms, patient history, and a comprehensive evaluation to identify the contributions of multiple pain mechanisms. Indeed, no specific screening or diagnostic tools currently exist for mixed pain, making it challenging for physicians to accurately identify and define this condition in patients with complex pain profiles [12].

Given these considerations, we would propose a comprehensive literature review combined with bibliometric analysis, which emerges as a valuable tool for enhancing understanding of the topic. Bibliometric analyses are statistical techniques used to quantitatively assess academic literature. The benefits of bibliometric analyses include identifying leading authors, influential institutions, and significant research topics, which help to trace the development of scientific knowledge [13]. Additionally, our analysis aims to uncover gaps in the existing research landscape, offering valuable insights that can inform and guide future investigations and studies.

## Methods

### Search Strategy and Data Collection

The methodology of our study follows standard procedures commonly used in bibliometric studies [14, 15]. Briefly, bibliometrics involves using quantitative analysis to assess academic literature, providing insights into research trends, influential authors, and the evolution of scientific knowledge. The process begins with the selection of a reliable database such as Web of Science to gather relevant publications through specific search queries. After data collection, the dataset is cleaned to remove duplicates and irrelevant entries, ensuring accuracy. The refined data is then analysed to identify patterns, measure impact, and explore the development of research within the selected field [16]. To ensure consistency and accuracy in the findings, a single database is utilized, which helps prevent data redundancy and overlap that could occur when using multiple sources.

A comprehensive review of global literature on mixed pain was conducted using the Web of Science Core Collection (WoSCC) online database. The search strategy employed the following search terms: “mixed” (All Fields) AND “pain” (All Fields). To ensure a focus on scientifically rigorous publications, the search was restricted to English-language sources. All relevant data were extracted on February 26, 2024, and exported in both TXT format for “full records and references” and Microsoft Excel (.xlsx) format. Bibliometric indicators, such as journals quartile rankings (Q) and impact factors (IF), were obtained from the Journal Citation Reports™ (Clarivate, 2022). Titles and abstracts were independently screened by three authors (R.G., M.R. and M.M.), who also reviewed the full-text versions of the articles. No relevant papers were excluded during this process. Cross-checking was performed, and any disagreements were resolved through discussions with the first and last authors (M.L.G.L., G.V).

### Data Processing and Analysis

The quantitative analysis of the number of papers and citations was conducted using Citation Report from Clarivate Analytics, while VOSviewer (Leiden University, Leiden, the Netherlands, version 1.6.20) was used to perform the network analysis. VOSviewer is a software tool designed for creating and visualizing bibliometric networks. It works by analyzing data from scientific publications, such as co-authorship, co-citation, and keyword co-occurrence, to generate maps that visually represent relationships between authors, institutions, or research topics [17]. These maps help to identify patterns, trends, and clusters within the scientific literature, making it easier to understand the inner structure and dynamics of research fields. Lastly, a manual review was performed to extract bibliometric data, such as journal quartiles (Q) and impact factors (IF), as well as to determine the types of documents included in the analysis. The Dickey-Fuller test was used to determine whether the time series data for the number of publications and citations is stationary over time, or if there is a unit root indicating the presence of a trend that drives the data away from its mean value [18]. The average annual percent change (APC) in the number of publications over time was calculated to assess the overall trend and rate of growth or decline in publication output. It is a statistical measure used to quantify the average rate of change over a specific period and was calculated by fitting a linear regression model to the logarithm of the annual publication counts. Join-point regression analysis was performed to identify points in time where there were significant changes in the trend, allowing for a more detailed understanding of shifts in the data over the study period [19]. R software v4.3.2 (R Foundation for Statistical Computing, Vienna, Austria, www.r-project.org) was used for the analyses.

## Results

**Fig. 1:**
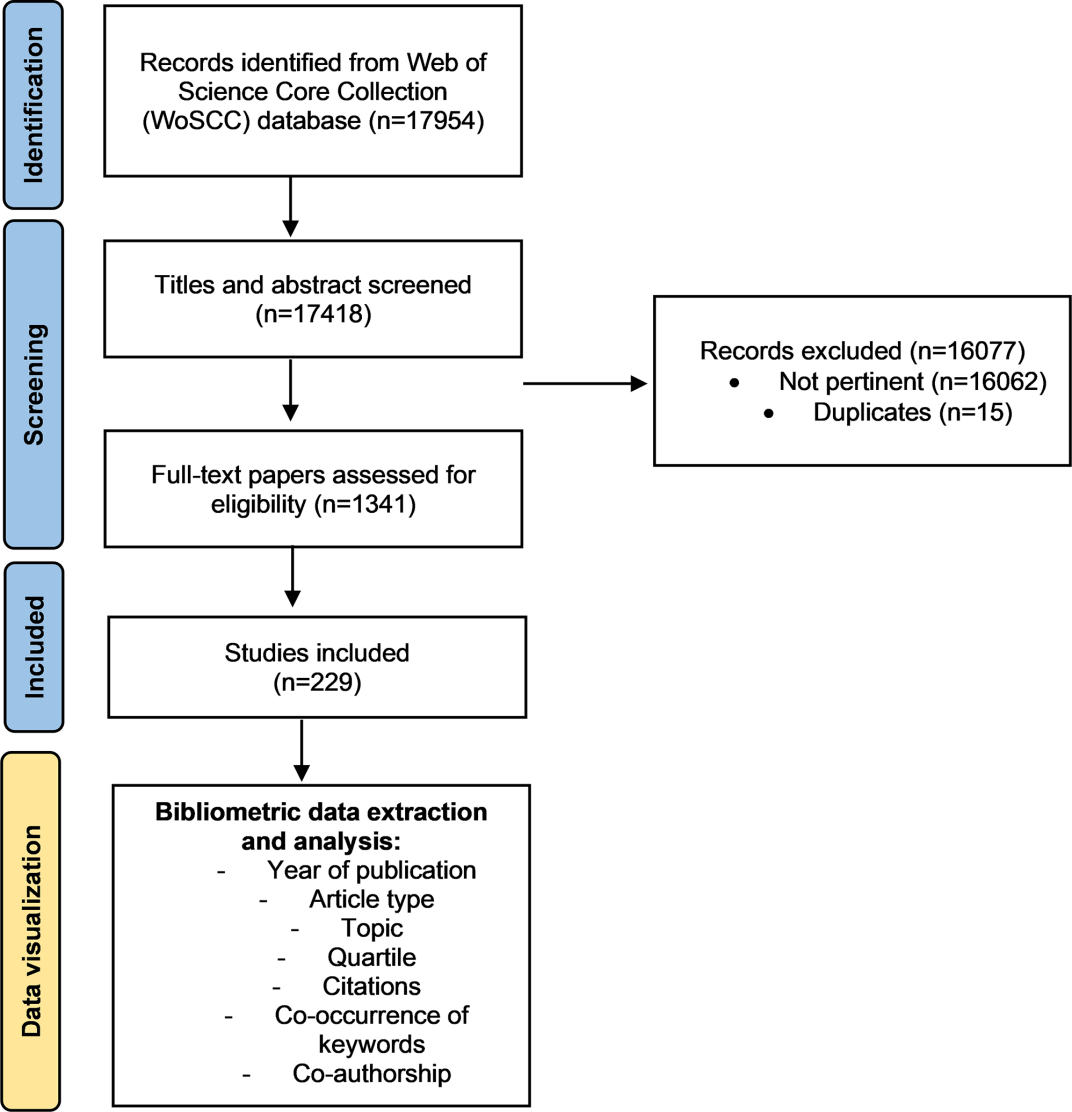
Flowchart of the study

By implementing the screening strategy, 17,954 records were initially identified from the WoSCC database, covering the period from 1993 to 2024. After excluding duplicates and non-pertinent studies, 1341 papers were evaluated for eligibility, leading to the inclusion of 229 studies deemed pertinent for analysis (Fig. 1). The selected studies underwent bibliometric data extraction, focusing on various aspects such as the year of publication, article type, research topic, quartile ranking, citations, keyword co-occurrence, and co-authorship patterns [20]. Co-occurrence of keywords is defined as the interconnections between keywords of different studies. Co-authorship analysis reflects the collaborative relationships between researchers while co-authorship between countries highlights international collaborations [21].

**Fig. 2:**
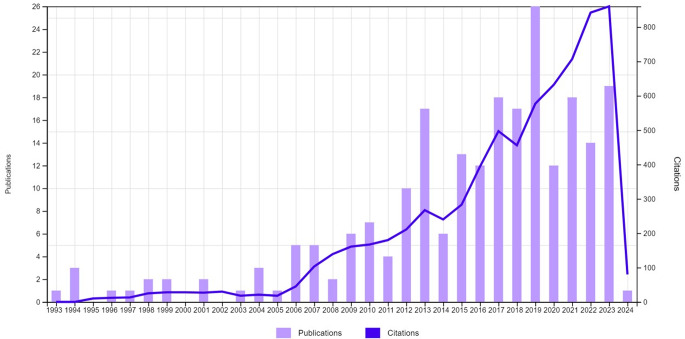
Annual publications and citations distribution

According to the Citation Report from WoSCC, over the whole study period (1993–2024), a progressive increase in the number of publications and distributions were observed (Fig. 2).

**Fig. 3:**
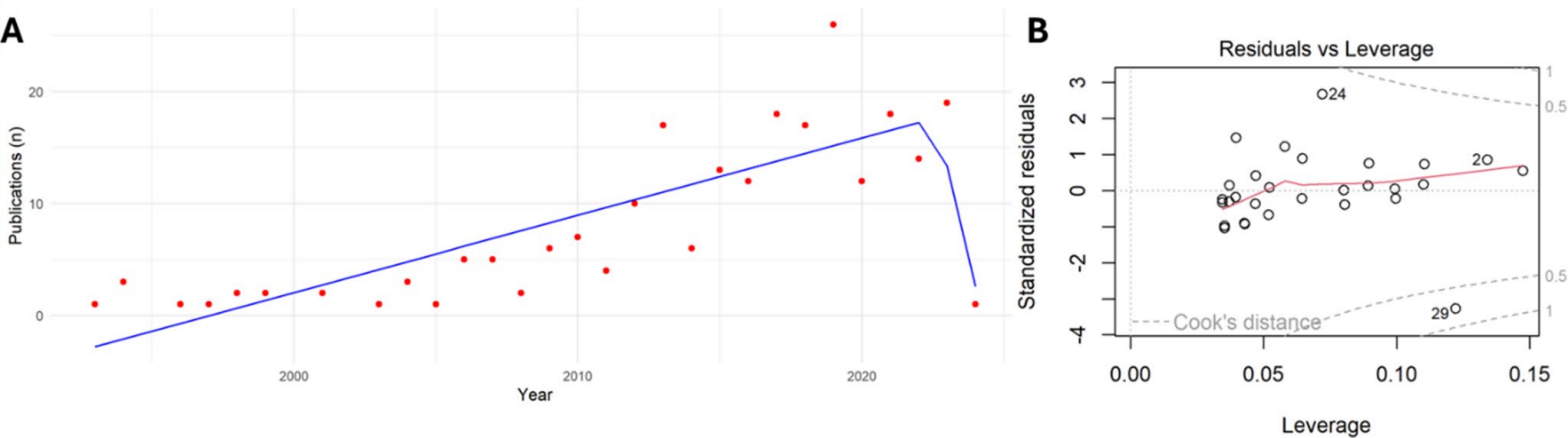
**A**) Analysis of the annual trend (join-point analysis) for the study period (1993–2024). **B**) Residuals versus leverage plot. The y-axis represents standardized residuals, which are the differences between observed and predicted values, standardized by the standard deviation of the residuals. The x-axis represents leverage, which measures how far an observation is from the mean of the predictor variable. The grey curved lines represent the Cook’s Distance, a measure of the influence of each data point on the regression coefficients. Points outside the Cook’s distance lines are considered influential, meaning they have a significant impact on the regression model. In this plot, the labelled points “2”, “24”, and “29” refers to the years 2017-2019-2023, indicate points with high leverage or high residuals, suggesting they could have a strong influence on the model

A relevant upward trend was observed over the time (Dickey-Fuller test, *p* = 0.64). A linear regression model revealed an ongoing rise in the number of publications on this topic (y = 0,57*x – 1135; *p* < 0.0001). The annual trend analysis (join-point analysis) identified two distinct segments with varying slopes (Fig. 3A). During the period from 1993 to 2023, there was a significant increase in the APC (APC = 4.9, slope of 69.1). In contrast, the period from 2023 to February 2024 showed a marked decrease in the APC, with a slope of -358.70, reflecting a reduced trend in the number of publications. This decline is directly attributable to the short observation period, as the data were extracted in the early months of 2024. Therefore, it is advisable to omit the APC calculation for the year 2024 due to the limited data available. As outlined by the residuals versus leverage plot (Fig. 3B) to highlight the influence of individual data points on the linear regression model, three years (2017-2019-2023) pulled the regression line upward.

Most documents were published in highly ranked Q1 journals as research articles (77%) or review articles (19%). In Table 1, we present the journals with the highest number of published papers, highlighting the top sources contributing to the literature in this field.

**Table 1:** Top journals by number of published papers. The table lists the journals that have published the most papers on the topic of mixed pain

Journal name	Documents *n* = 229, (%)	5-Year Journal Impact factor	Category quartile
Pain Practice	12 (5.2%)	2.8	Q2
Clinical Journal of pain	8 (3.5%)	3.5	Q2
Current medical research and opinion	7 (3%)	2.5	Q2
Journal of Pain	7 (3%)	5	Q1
Pain	7 (3%)	7.1	Q1
European journal of pain	5 (2.2%)	3.8	Q1
Journal of pain research	5 (2.2%)	2.8	Q2
Pain medicine	5 (2.2%)	3.2	Q1
Cochrane database of systematic reviews	4 (1.7%)	9.9	Q1
Journal of pain and symptom management	4 (1.7%)	3.8	Q1
Pain physician	4 (1.7%)	3.1	Q2
Applied Sciences-Basel	3 (1.3%)	2.7	Q2
Minerva medica	3 (1.3%)	Unavailable due to anomalous citation patterns observed in 2023	Q1
Neuromodulation	3 (1.3%)	3.5	Q2
Pain and Therapy	3 (1.3%)	4.2	Q1
Plos one	3 (1.3%)	3.3	Q1

**Fig. 4:**
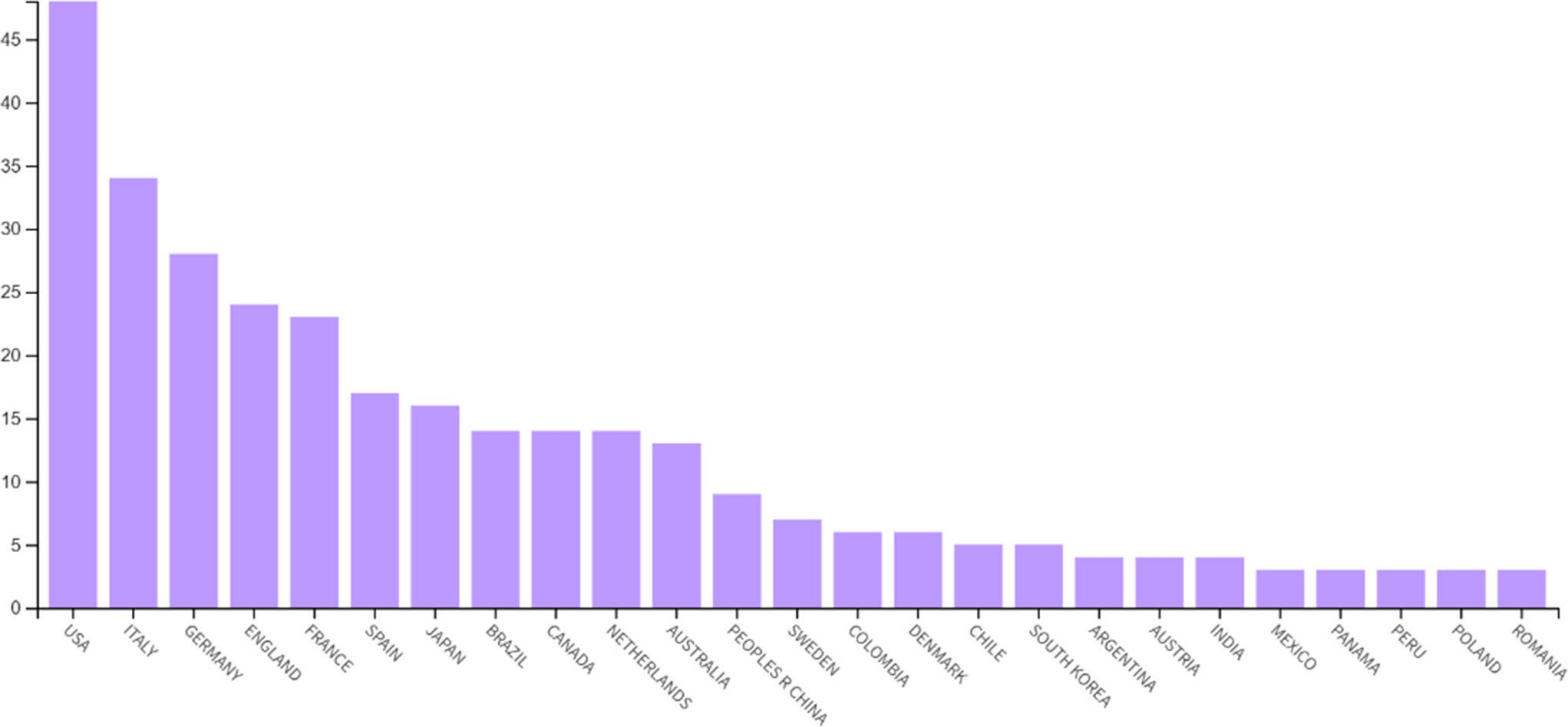
Distribution of Published Papers by Country: number of papers on mixed pain published by various countries, with the USA, Italy, and Germany leading the contributions

Figure 4 illustrates the distribution of published papers on the topic of mixed pain across various countries. The USA (21%), Italy (15%), and Germany (12%) were the leading contributors, accounting for the majority of publications.

**Fig. 5:**
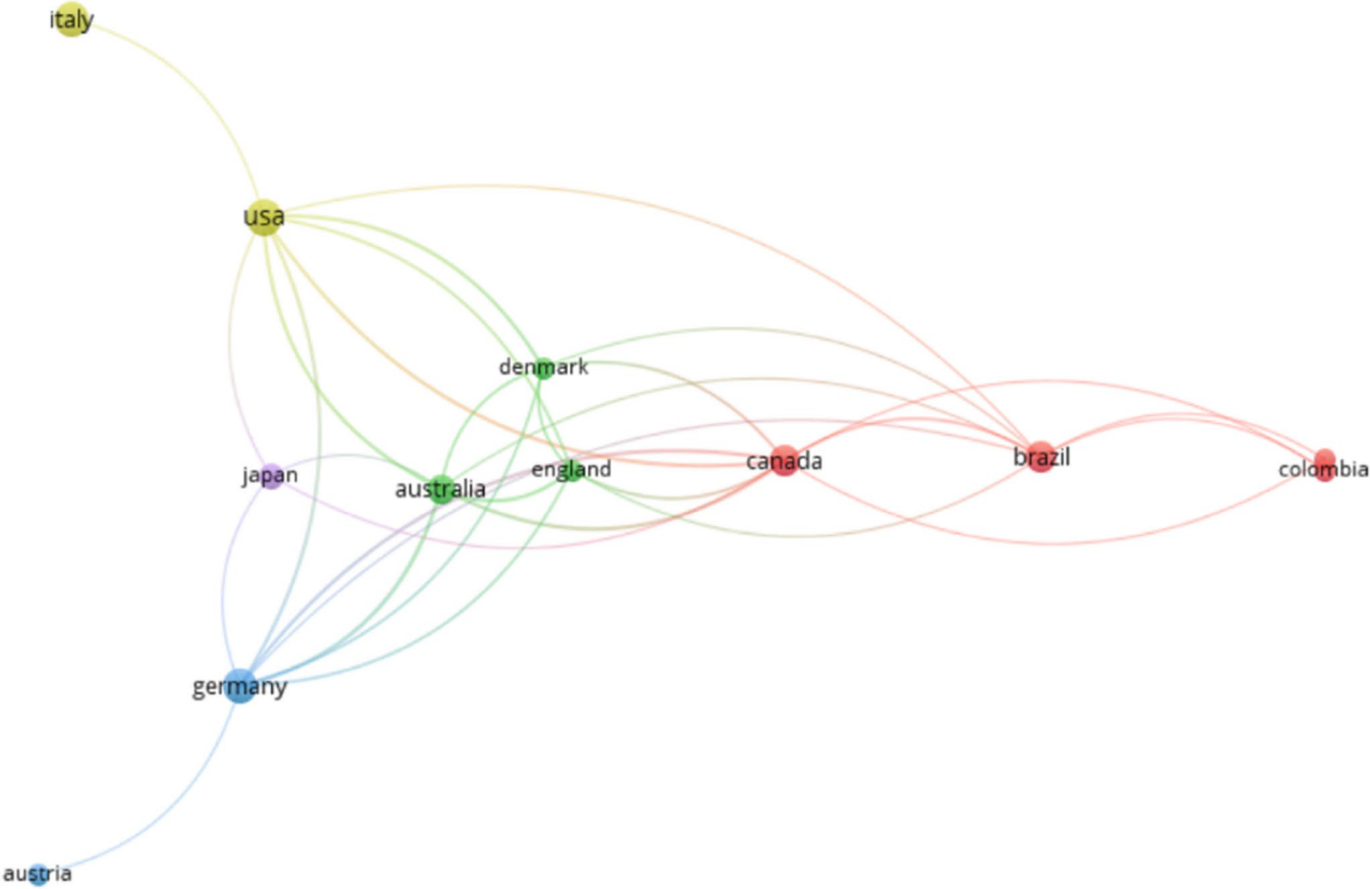
Co-authorship analysis for countries. In the network, the nodes represent countries, and the lines between them indicate collaborative relationships in co-authored publications. The thickness and intensity of the lines reflect the strength or frequency of these collaborations. A total of 11 countries met the threshold, with a minimum of 5 documents per country

The co-authorship analysis for countries was conducted with a minimum threshold of five documents per country to evaluate the collaboration between different countries in publishing papers on the topic of mixed pain (Fig. 5). An amount of 11 countries met the threshold with a total of 65 documents and a total link strength of 78. The time-based analysis of co-authorship revealed no strong connections or organized structure among countries. However, the USA emerged as a central hub in this network (8 documents, 12 links), showing connections with other countries, including Italy, Canada, and Brazil. Germany, England, and Australia also demonstrate collaborative ties, albeit to a lesser extent than the USA. Despite its large number of published papers on mixed pain, Italy appeared relatively isolated, with connections limited to the USA.

**Fig. 6:**
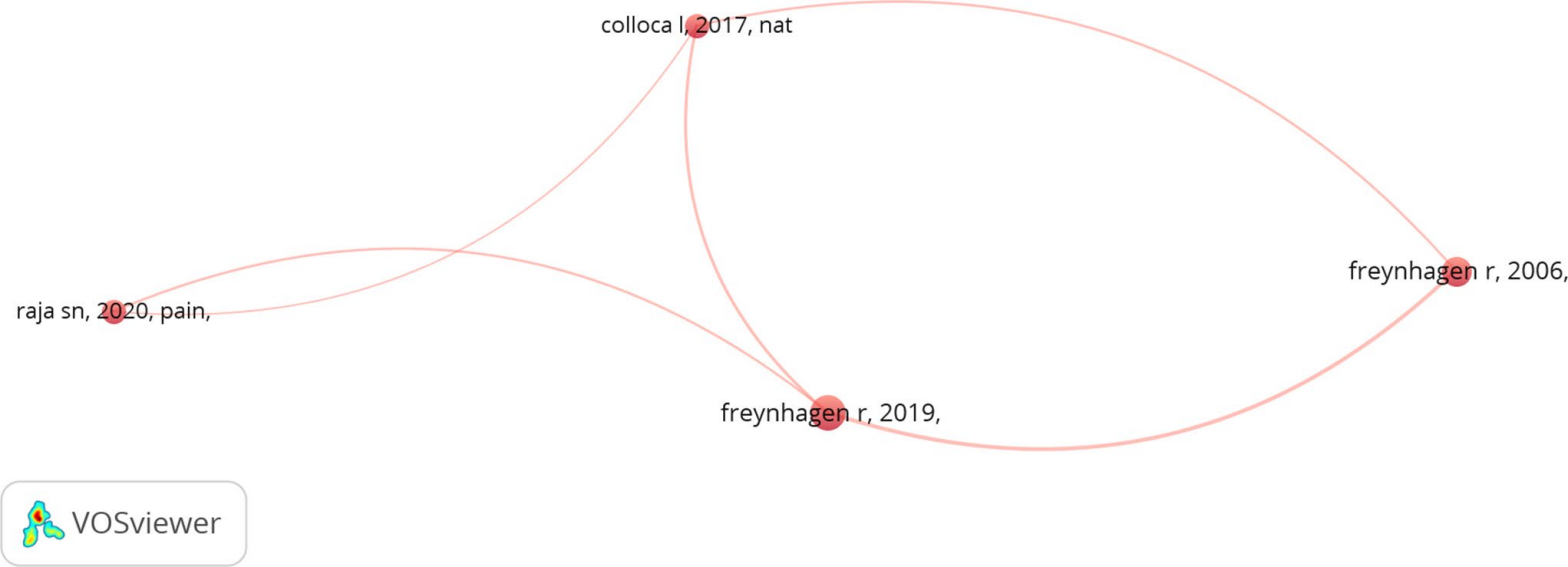
Co-authorship analysis for authors. This figure illustrates the citation relationships between influential papers in the field of mixed pain research. The nodes represent individual publications, and the lines between them represent citation links, indicating that one paper has cited another. The thickness of the lines suggests the strength or frequency of these citation connections. The figure highlights the interconnection between different authors such as Freynhagen et al. (2006, 2019), Colloca et al. (2017), and Raja et al. (2020)

**Fig. 7:**
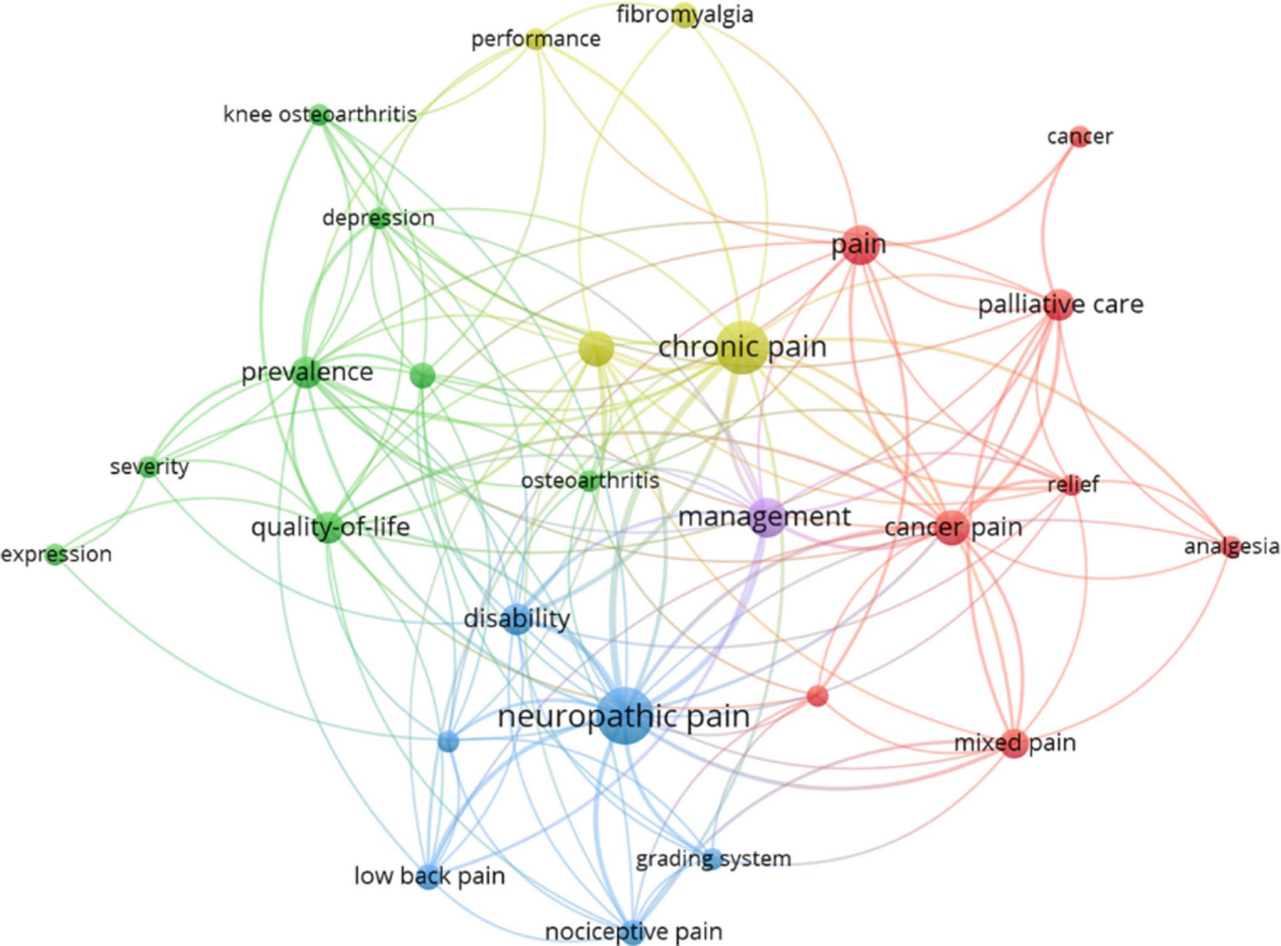
Keywords co-occurrence analysis illustrating the relationships and connections between frequently used terms in the field of mixed pain. The nodes represent individual keywords, with larger nodes indicating terms that occur more frequently. The lines connecting the nodes represent co-occurrences of these keywords within the same publications, with thicker lines indicating stronger associations

The map of Co-authorship for authors conducted with a cutoff of at least two documents for a single author revealed no clusters of strong collaboration between authors. In this network, the paper by Freynhagen et al. (2006) [22] appears as a significant node, being cited by other key publications, including subsequent works by Freynhagen (2019) [23]. The network also highlights connections between other influential papers, such as those by Colloca et al. (2017) [24] and Raja et al. (2020) [1] (see Fig. 6 and 7).

The bibliometric analysis focused on keywords that appeared more than five times in the database. Of the 411 keywords, 25 reached the threshold. Five clusters were collected. The most frequently used keywords were “chronic pain,” “neuropathic pain,” “management,” “quality of life,” and “pain,” indicating their prominence and importance within this topic. These clusters of keywords highlight distinct yet interconnected areas of study, such as the link between chronic pain and neuropathic pain, the connection between chronic pain and management, and the association between neuropathic pain and disability.

## Discussion

A bibliometric analysis offers a valuable opportunity to identify potential gaps in the literature, to quantitatively evaluate academic literature, providing insights into research trends, influential authors which can, in turn, guide future research efforts [25]. To the best of our knowledge, this is the first bibliometric analysis focused on the topic of mixed pain. Therefore, the results from this study could be highly beneficial for shaping and ultimately directing further research in the field.

Given the complexity and clinical relevance of mixed pain, our study aimed to conduct a comprehensive bibliometric analysis to enhance understanding of this topic. Our analysis revealed a progressive increase in the number of publications on mixed pain from 1993 to 2024, indicating growing interest in this area. This upward trend was particularly pronounced from 1993 to 2023, with a significant APC in the number of publications.

Concerning the quality of the journals, most documents were published in highly ranked Q1 journals, and the journal “Pain Practice” published 12 papers, followed by “Clinical Journal of Pain” that published 8 articles. Analyzing sources is beneficial for authors as it aids in searching the literature and selecting an appropriate journal for submitting their articles. The progressive increase in the number of citations over time highlights the visibility of these articles, although this increase is not necessarily indicative of their quality [26]. However, it reflects the interest in the topic of mixed pain, as evidenced by the types of published papers: research articles (77%) and review articles (19%). These factors suggest an overall high level of research quality.

The co-authorship analysis for countries highlighted the central role of certain countries in mixed pain research. The USA emerged as a dominant contributor, accounting for 21% of the total publications, followed by Italy and Germany. The co-authorship analysis for countries further emphasized the USA acting as a central hub in international collaborations. Unfortunately, despite its significant publication output, Italy appeared relatively isolated, with limited connections to other countries. As previously observed in the publication patterns of Italian sociologists, this isolation effect may be linked to the distorting influence of institutional signals on publication choices. Italian sociologists have been known to publish more frequently in journals considered influential for assessment purposes, but often without engaging in an international collaborative network [27]. This suggests potential opportunities for enhancing international collaboration in mixed pain research, particularly involving countries with high research output (USA, Italy, Canada, Brazil, Germany, England, and Australia). In addition to geographical trends, the analysis of co-authorship among individual authors revealed the influence of key publications in shaping the field. The works of Freynhagen et al. [22, 23], were identified as significant nodes within the citation network, indicating their impact on subsequent research. In fact, their publication of 2019 discussed the concept of “mixed pain”, which is increasingly used to describe clinical scenarios such as low back pain, cancer pain, and postsurgical pain, where different types of pain (nociceptive, neuropathic, and nociplastic) overlap and cause pain in the same area [23]. They highlighted whether mixed pain is due to these mechanisms operating simultaneously or if it represents a distinct pathophysiological process. In fact, the diagnosis of mixed pain relies on clinical judgment rather than formal screening or diagnostic criteria, which poses challenges for pain physicians. The authors proposed also a methodical approach for evaluating patients with acute, subacute, or chronic pain, including nine key questions to help clinicians identify the predominant pain mechanisms [28]. However, despite its extensive use in the literature, the term “mixed pain” has never been formally defined even if it is increasingly recognized and accepted by pain clinicians to describe these complex cases.

The keyword analysis in our bibliometric study highlighted the importance of terms such as “neuropathic pain,” “management,” and “quality of life” within the field of mixed pain research. “Neuropathic pain” emerged as a central focus, reflecting also its role as a component of mixed pain. This highlights the ongoing interest in understanding and addressing the complex mechanisms underlying neuropathic pain, which is notoriously difficult to treat [24]. A significant portion of our current knowledge about the causes of neuropathic pain and the identification of drug targets comes from studies examining the effects of peripheral nerve injury in rodent models [29, 30]. While traditional animal models have uncovered critical aspects of pain etiology, including peripheral and central sensitization and various molecular and cellular mechanisms, they fall short in capturing the full range of disease states or injuries that lead to neuropathic pain in clinical settings. Consequently, translating findings from the lab to effective clinical treatments has achieved only limited success [31, 32]. There is an urgent need to find new treatments for neuropathic pain and combination therapies such as drug-drug co-crystallization may represent a potential therapeutic approach to target the multiple mechanisms of neuropathic pain [33].

Directly related to mixed pain and neuropathic pain is the keyword “management”. This underscores the need for effective strategies to treat mixed pain. As highlighted in the Latin American consensus meeting on mixed pain, the emphasis was placed on the importance of multidisciplinary approaches in achieving optimal patient outcomes [11]. Moreover, the Latin American consensus highlighted the potential role of vitamins B1, B6, and B12, whether used alone or as an adjunct to other analgesic drugs, in the management of mixed pain, based on positive findings from both preclinical and clinical studies on nociceptive and neuropathic pain [34–38]. Unfortunately, despite the promising potential of this approach, there are currently no studies specifically focused on exploring the role of B vitamins in the management of mixed pain.

Additionally, the frequent occurrence of “quality of life” as keyword indicates a growing recognition of the broader impact of pain on patients’ overall well-being. The evaluation of the impact of pain on quality of life was clearly recommended by the IASP [1] and explored in neuropathic and cancer pain [39]. However, no specific studies have addressed the quality of life in mixed pain patients compared to those with nociceptive, neuropathic or nociplastic pain. This gap in the research highlights the need for future studies to develop a formal definition of mixed pain, explore the quality of life in individuals with mixed pain, and investigate any unique characteristics that may differentiate them from other pain populations.

The keyword analysis provided a valuable method for identifying key themes in mixed pain and establishing a framework of relevant documents within this field of research. Unfortunately, only five clusters were identified, indicating a lack of cohesion among keywords, which are dispersed across a wide range of scientific papers. This suggests that the research on mixed pain is still fragmented and lacks unified focus.

### Limitations

This analysis has several limitations. The search and methods were confined to articles extracted and analyzed from the WoSCC database, which may have led to the exclusion of articles available in other databases (e.g., Scopus or PubMed) or in the grey literature. Although WoSCC was chosen due to its widespread use in bibliometric studies [40], this limitation should be considered when interpreting the results. Furthermore, as Gasparyan et al. [41] recently suggested, the significance of article metrics should be evaluated while considering confounding factors, such as citation patterns and social media engagement across different countries and academic disciplines. In fact, external factors that are not fully understandable can influence citations, keywords and authors patterns. Another limitation of our study is that we did not perform a Price index analysis. The Price index, based on Price’s Law, is widely used to assess the growth trends of scientific outputs within a specific country or academic discipline [42]. It helps determine whether the increase in publications follows an exponential or linear pattern. However, given our findings regarding the international distribution of research on mixed pain, where research efforts are fragmented and collaborative networks are limited, we opted not to perform this analysis. The diverse and uneven contributions from different countries in the field of mixed pain could have led to skewed results, making the Price index less applicable in this context. Another potential limitation is the search string we used for the bibliometric analysis, as it may have been incomplete or not exhaustive in capturing all published articles on this topic. However, the large number of articles initially extracted from WoSCC, many of which were later excluded due to being non-pertinent or duplicates, suggests that our search strategy was effective in comprehensively identifying relevant indexed papers. This process likely ensured that most of the pertinent literature was included in our analysis. Finally, the rise in publications found in our analysis should be considered in the context of the overall growth in publications indexed in WoSCC and also the increase in mixed pain research may be influenced by this general trend [43]. Consequently, this analysis should be updated periodically.

### Suggestions for Future Research

The primary goal of a bibliometric study is to provide guidance and recommendations for future research. Given the current landscape of mixed pain research, several key areas warrant further exploration. Firstly, there is an urgent need for a formal definition of mixed pain, along with the development of diagnostic criteria and specific guidelines to aid clinicians in accurately identifying and managing this complex condition. Additionally, due to the limited group characteristics observed in cooperative networks, it appears that authors tend to publish independently. These results highlight the need to strengthen international partnerships and collaboration to foster more balanced and cohesive progress in the field of mixed pain. Future studies should also be focused on investigating the role of vitamins B1, B6, and B12 in mixed pain management, building on the promising results seen in nociceptive and neuropathic pain. The quality of life and the presence of comorbidities in patients with mixed pain remain underexplored, particularly in comparison to those with purely nociceptive or neuropathic pain. Research in this area could uncover unique characteristics that may guide more tailored treatment approaches. Moreover, enhancing international collaboration is essential to address the current fragmentation in the field, as evidenced by the dispersed keyword clusters and limited cooperative networks. By strengthening partnerships and fostering more cohesive research efforts, the global understanding and treatment of mixed pain can be significantly advanced.

## Conclusions

Our bibliometric analysis provides a comprehensive overview of the research landscape on mixed pain, highlighting key trends, influential contributors, and potential gaps in the literature. The findings from this study can inform future research efforts, guiding the development of more effective diagnostic tools and treatment strategies for mixed pain. Moreover, the identification of central themes and collaborative networks underscores the importance of a multidisciplinary approach to addressing this complex condition. Future studies should aim to expand on these findings by incorporating a broader range of data sources, extending the follow-up period, and exploring the economic implications of mixed pain management. Finally, our data underscore the urgent need for a formal definition of mixed pain, as well as the development of diagnostic criteria and specific guidelines for its identification and treatment. In fact, the absence of a formal definition and diagnostic criteria for mixed pain creates challenges in accurately identifying and treating patients with overlapping nociceptive, neuropathic and nociplastic pain. Establishing clear guidelines is essential to improve clinical outcomes and provide standardized care for this complex condition.

## Data Availability

The data used in this study were retrieved from the Web of Science Core Collection (WoSCC) database. Data supporting the findings of this study are available upon reasonable request from the corresponding author.

## Abbreviations

IASP: International Association for the Study of Pain
WoSCC: Web of Science Core Collection
APC: Average annual percent change
Q1: First quartile journals (most prestigious journals)

## References

[1] Raja SN, Carr DB, Cohen M, Finnerup NB, Flor H, Gibson S, et al. The revised International Association for the study of Pain definition of pain: concepts, challenges, and compromises. Pain. 2020;161:1976–82. 32694387 10.1097/j.pain.0000000000001939 PMC7680716

[2] Fitzcharles M-A, Cohen SP, Clauw DJ, Littlejohn G, Usui C, Häuser W. Nociplastic pain: towards an understanding of prevalent pain conditions. Lancet Lond Engl. 2021;397:2098–110. 10.1016/S0140-6736(21)00392-5 34062144

[3] Scholz J, Finnerup NB, Attal N, Aziz Q, Baron R, Bennett MI, et al. The IASP classification of chronic pain for ICD-11: chronic neuropathic pain. Pain. 2019;160:53–9. 30586071 10.1097/j.pain.0000000000001365 PMC6310153

[4] Terminology | International Association for the Study of Pain [Internet]. Int. Assoc. Study Pain IASP. [cited 2024 Aug 18]. Available from: https://www.iasp-pain.org/resources/terminology/

[5] Kosek E, Cohen M, Baron R, Gebhart GF, Mico J-A, Rice ASC, et al. Do we need a third mechanistic descriptor for chronic pain states? Pain. 2016;157:1382–6. 26835783 10.1097/j.pain.0000000000000507

[6] Ross E. Moving towards rational pharmacological management of pain with an improved classification system of pain. Expert Opin Pharmacother. 2001;2:1529–30. 11825296 10.1517/14656566.2.10.1529

[7] Trouvin A-P, Perrot S. New concepts of pain. Best Pract Res Clin Rheumatol. 2019;33:101415. 31703792 10.1016/j.berh.2019.04.007

[8] Picelli A, Buzzi MG, Cisari C, Gandolfi M, Porru D, Bonadiman S et al. Headache, low back pain, other nociceptive and mixed pain conditions in neurorehabilitation. Evidence and recommendations from the Italian Consensus Conference on Pain in Neurorehabilitation. Eur J Phys Rehabil Med. 2016;52:867–80. 27830925

[9] Ibor PJ, Sánchez-Magro I, Villoria J, Leal A, Esquivias A. Mixed Pain can be discerned in the primary care and Orthopedics settings in Spain: a large cross-sectional study. Clin J Pain. 2017;33:1100–8. 28244943 10.1097/AJP.0000000000000491

[10] Latina R, De Marinis MG, Giordano F, Osborn JF, Giannarelli D, Di Biagio E, et al. Epidemiology of Chronic Pain in the Latium Region, Italy: a cross-sectional study on the clinical characteristics of patients attending Pain clinics. Pain Manag Nurs off J Am Soc Pain Manag Nurses. 2019;20:373–81. 10.1016/j.pmn.2019.01.005 31103514

[11] Fernandez-Fairen M, Calderón-Ospina CA, Chen J, Duarte Vega M, Fernández-Villacorta F, Gómez-García F, et al. A latin American consensus meeting on the essentials of mixed pain. Curr Med Res Opin. 2023;39:451–66. 36772818 10.1080/03007995.2023.2177401

[12] Guillén-Núñez M, del Juárez-Lemus R, Hernández-Porras AM. Hernández-Rodríguez D. Current perspective in mixed Pain. J Drug Deliv Ther. 2024;14:170–3.

[13] Donthu N, Kumar S, Mukherjee D, Pandey N, Lim WM. How to conduct a bibliometric analysis: an overview and guidelines. J Bus Res. 2021;133:285–96.

[14] Manoj Kumar L null, George RJ. P S A. Bibliometric Analysis for Medical Research. Indian J Psychol Med. 2023;45:277–82. 10.1177/02537176221103617 PMC10159556 37152388

[15] Ninkov A, Frank JR, Maggio LA. Bibliometrics: methods for studying academic publishing. Perspect Med Educ. 2022;11:173–6. 34914027 10.1007/s40037-021-00695-4 PMC9240160

[16] Passas I. Bibliometric analysis: the main steps. Encyclopedia. 2024;4:1014–25.

[17] van Eck NJ, Waltman L. Software survey: VOSviewer, a computer program for bibliometric mapping. Scientometrics. 2010;84:523–38. 20585380 10.1007/s11192-009-0146-3 PMC2883932

[18] Monroy SE, Diaz H. Time series-based bibliometric analysis of the dynamics of scientific production. Scientometrics. 2018;115:1139–59.

[19] Gillis D, Edwards BPM. The utility of joinpoint regression for estimating population parameters given changes in population structure. Heliyon. 2019;5:e02515. 31768426 10.1016/j.heliyon.2019.e02515 PMC6872810

[20] Wu H, Yamaguchi A. Semantic web technologies for the big data in life sciences. Biosci Trends. 2014;8:192–201. 25224624 10.5582/bst.2014.01048

[21] Fonseca B, de PFE, Sampaio RB, Fonseca MV, de Zicker A. Co-authorship network analysis in health research: method and potential use. Health Res Policy Syst. 2016;14:34. 27138279 10.1186/s12961-016-0104-5 PMC4852432

[22] Freynhagen R, Baron R, Tölle T, Stemmler E, Gockel U, Stevens M, et al. Screening of neuropathic pain components in patients with chronic back pain associated with nerve root compression: a prospective observational pilot study (MIPORT). Curr Med Res Opin. 2006;22:529–37. 16574036 10.1185/030079906X89874

[23] Freynhagen R, Parada HA, Calderon-Ospina CA, Chen J, Rakhmawati Emril D, Fernández-Villacorta FJ, et al. Current understanding of the mixed pain concept: a brief narrative review. Curr Med Res Opin. 2019;35:1011–8. 30479161 10.1080/03007995.2018.1552042

[24] Colloca L, Ludman T, Bouhassira D, Baron R, Dickenson AH, Yarnitsky D, et al. Neuropathic pain. Nat Rev Dis Primer. 2017;3:17002. 10.1038/nrdp.2017.2 PMC5371025 28205574

[25] Hashem EAR, Md Salleh NZ, Abdullah M, Ali A, Faisal F, Nor RM. Research trends, developments, and future perspectives in brand attitude: a bibliometric analysis utilizing the Scopus database (1944–2021). Heliyon. 2023;9:e12765. 36685379 10.1016/j.heliyon.2022.e12765 PMC9850049

[26] Huang Y-J, Cheng S, Yang F-Q, Chen C. Analysis and visualization of Research on resilient cities and communities based on VOSviewer. Int J Environ Res Public Health. 2022;19:7068. 35742316 10.3390/ijerph19127068 PMC9223032

[27] Akbaritabar A, Bravo G, Squazzoni F. The impact of a national research assessment on the publications of sociologists in Italy. Sci Public Policy. 2021;48:662–78.

[28] Freynhagen R, Rey R, Argoff C. When to consider mixed pain? The right questions can make a difference! Curr Med Res Opin. 2020;36:2037–46. 33012210 10.1080/03007995.2020.1832058

[29] Bouali-Benazzouz R, Landry M, Benazzouz A, Fossat P. Neuropathic pain modeling: focus on synaptic and ion channel mechanisms. Prog Neurobiol. 2021;201:102030. 33711402 10.1016/j.pneurobio.2021.102030

[30] Decosterd I, Woolf CJ. Spared nerve injury: an animal model of persistent peripheral neuropathic pain. Pain. 2000;87:149–58. 10924808 10.1016/S0304-3959(00)00276-1

[31] Ghazisaeidi S, Muley MM, Salter MW. Neuropathic Pain: mechanisms, sex differences, and potential therapies for a global problem. Annu Rev Pharmacol Toxicol. 2023;63:565–83. 36662582 10.1146/annurev-pharmtox-051421-112259

[32] Soliman N, Kersebaum D, Lawn T, Sachau J, Sendel M, Vollert J. Improving neuropathic pain treatment - by rigorous stratification from bench to bedside. J Neurochem [Internet]. 2023 [cited 2024 Aug 17]; Available from: 10.1111/jnc.15798 10.1111/jnc.15798 36852505

[33] Boccella S, De Filippis L, Giorgio C, Brandolini L, Jones M, Novelli R, et al. Combination drug therapy for the management of Chronic Neuropathic Pain. Biomolecules. 2023;13:1802. 38136672 10.3390/biom13121802 PMC10741625

[34] Gazoni FM, Malezan WR, Santos FC. B Complex vitamins for analgesic therapy. Rev Dor [Internet]. 2016 [cited 2024 Aug 17];17. Available from: http://www.scielo.br/scielo.php?script=sci_arttext%26pid=S1806-00132016000100052%26lng=pt%26nrm=iso%26tlng=pt

[35] Yu C-Z, Liu Y-P, Liu S, Yan M, Hu S-J, Song X-J. Systematic administration of B vitamins attenuates neuropathic hyperalgesia and reduces spinal neuron injury following temporary spinal cord ischaemia in rats. Eur J Pain Lond Engl. 2014;18:76–85. 10.1002/j.1532-2149.2013.00390.x 24038589

[36] Xu J, Wang W, Zhong X-X, Feng Y-W, Wei X-H, Liu X-G. Methylcobalamin ameliorates neuropathic pain induced by vincristine in rats. Mol Pain. 2016;12:1744806916657089. 27306413 10.1177/1744806916657089 PMC4956006

[37] Song X-S, Huang Z-J, Song X-J. Thiamine suppresses thermal hyperalgesia, inhibits hyperexcitability, and lessens alterations of sodium currents in injured, dorsal root ganglion neurons in rats. Anesthesiology. 2009;110:387–400. 19194165 10.1097/ALN.0b013e3181942f1e

[38] Zhang M, Han W, Zheng J, Meng F, Jiao X, Hu S, et al. Inhibition of hyperpolarization-activated Cation Current in Medium-Sized DRG neurons contributed to the Antiallodynic Effect of Methylcobalamin in the rat of a Chronic Compression of the DRG. Neural Plast. 2015;2015:197392. 26101670 10.1155/2015/197392 PMC4460234

[39] Madariaga Muñoz MC, Villegas Estévez F, Jiménez López AJ. Cabezón Álvarez A, Soler López B. evaluation of quality of life and satisfaction of patients with Neuropathic Pain and breakthrough Pain: economic impact based on quality of life. Pain Res Treat. 2018;2018:5394021. 30254760 10.1155/2018/5394021 PMC6145165

[40] Pranckutė R. Web of Science (WoS) and Scopus: the titans of Bibliographic Information in Today’s Academic World. Publications. 2021;9:12.

[41] Gasparyan AY, Yessirkepov M, Voronov AA, Maksaev AA, Kitas GD. Article-Level Metrics. J Korean Med Sci. 2021;36:e74. 33754507 10.3346/jkms.2021.36.e74 PMC7985291

[42] Huang X, Yang Z, Zhang J, Wang R, Fan J, Zhang H, et al. A bibliometric analysis based on web of Science: current perspectives and potential trends of SMAD7 in Oncology. Front Cell Dev Biol. 2021;9:712732. 35252215 10.3389/fcell.2021.712732 PMC8894759

[43] Bornmann L, Haunschild R, Mutz R. Growth rates of modern science: a latent piecewise growth curve approach to model publication numbers from established and new literature databases. Humanit Soc Sci Commun. 2021;8:1–15.38617731

